# Supplementary material for: The influence of stimulus duration on olfactory perception
Source: PLoS One. 2021 Jun 10;16(6):e0252931. doi: 10.1371/journal.pone.0252931 (PMC8191971; doi:10.1371/journal.pone.0252931)
Supplement: Attachment — Submitted filename: PLOS ONE Response to reviewers.docx [file pone.0252931.s008.docx]

**PLOS ONE Response to reviewers**

**Response to the editor**

1) **Editor:** In particular, both reviewers note that there is no positive control for the 10 ms stimulation, an issue that has to be addressed carefully.

Author response: However we didn’t include the data in the initial manuscript, we now included additional data and figures for 10 ms positive control (Line 265-273, Fig 5 G,H,I). Our additional data shows that mice can detect 10 ms light stimulation and avoiding the light zone when trained with foot shock and 10 ms light stimulation (P = 0.0005, n=4).

2) **Editor:** I also concur that the use of “olfactory identity” might not be appropriate.

Author response: Olfactory identity changed to olfactory perception

3) **Editor:** Stimulating the entire bulb hardly could be seen as conferring olfactory identity.

Author response: We did not stimulate the entire OB. We are stimulating only a few groups of glomeruli in the olfactory bulb to activate the OSNs to induce an odor response.

4) **Editor:** Since the authors permanently closed off one leg of the “Y” maze (as they call it) they might as well not call it a “Y” maze since it is not a Y maze. And avoid confusion.

Author response: We would prefer to keep it as “modified Y maze” to avoid the confusion with the supplementary video provided. We believe readers might get confused when comparing the video and the cartoon (Figure 1B). We have explained in the method section that one arm of the ‘Y’ maze is permanently closed, so the animals do not have access to the third arm at any point in the training or test session.

5) **Editor:** I might have missed it, but it was not clear to me how often during the testing mice were light-stimulated.

Author response: We have included additional line in the manuscript “The light stimulation was delivered in the Light zone each time when the mice completely entered that zone” (Line 117).

**Response to Reviewer 1**

1) **Reviewer1:** Please rephrase the title to something that is more descriptive of the actual experiments carried out and the conclusions that can be drawn.

Author response: Title changed to “The influence of stimulus duration on olfactory Perception”

2) **Reviewer1:** The authors need to better highlight the motivation to study the effect of stimulus duration in the context of olfaction and be more specific in the description of experiments. Where is channelrhodopsin expressed?

Author response: We have revised the abstract and included the location of channelrhodopsin expression (line 18).

3) **Reviewer1:** Line 27-28: Beyond the contribution of respiration, gaining tight control over the odour stimulus remains a challenge, please elaborate.

Author response: Added more explanation and citations (line 32-43).

4) **Reviewer1:** Line 28-29: The authors mention complex binding properties in the olfactory epithelium. Please include that natural odours rarely occur isolated and include the potential of antagonistic odour-odour interactions.

Author response: Included in the manuscript “The interaction of these complex odor mixtures and the activation of different OSNs shows antagonistic interactions among odorants” (line 35)

5) **Reviewer1:** Line 31: Please elaborate on the importance of precise timing and include studies that stimulated with actual odours instead of artificial light stimulation (e.g. Parabucki et al., 2019, Jordan et al., 2018).

Author response: Elaborated the importance of precise timing and included additional studies. (Please see line 46-52).

**6) Reviewer1:** Line 32: Please add references that provide excellent description of odour plume physics (e.g. by Vergassola, Celani, Crimaldi)

Author response: Elaborated and added additional references by Vergassola, Celani, Crimaldi. (Please see line 46-52).

7) **Reviewer1:** Line 34-35: While all these excellent studies suggest that temporal information is relevant for mammalian behaviour, direct evidence for this is still lacking. This highlights the need for carefully designed behavioural and physiological mammalian experiments. To provide context, the authors should cite some invertebrate studies (some are mentioned in the discussion) to underline the importance of temporal odour information.

Author response: Added more citation for invertebrate studies (reference 21-30, line 52).

8) **Reviewer1:** Line 39-40: In the context of temporal discrimination in bees the authors should include Szyszka et al., 2013

Author response: Added the study by Szyszka et al., 2013. Please see line 60.

9) **Reviewer1:** Line 45: The authors are correct in pointing out the critical effect of sniffing during odour sampling. They should mention that in many studies efforts are made to reduce variability in odour stimulation by triggering the stimulus during a certain phase of the respiration cycle.

Author response: Added new line (line 64)“To reduce the variability in odor stimulation, in many studies odor is delivered at a certain phase of the respiration cycle [33–37].”

10) **Reviewer1:** Line 48-49: Please mention that both uni- and bilateral stimulation paradigms were performed.

Author response: mentioned uni- and bilateral stimulation paradigms (line 67).

11) **Reviewer1:** Line 93: What is the time between the first training session and the test session, i.e. how many reinforcement sessions did the animals undergo?

Author response: One reinforcement session pe day. Please see line 114.

12) **Reviewer1:** Line 95: Why pair foot shock with only unilateral OB stimulation?

Author response: The aim of the unilateral pairing was to check the avoidance response during the ipsilateral and contralateral OB stimulation separately. We also performed foot shoch pairing with bilateral OB stimulation. Please see line 229.

13) **Reviewer1:** Line 98: Why choose ten light 50 ms pulses duration and 150 ms interval? There is no mention of the sniff cycle. While probably out of scope for a revised version of this manuscript, implanting a sniff cannula or use an implanted telemetric sensor would greatly advance the study and open up the opportunity to trigger light stimulation during specific phases of the respiration cycle and, thus allow to relate behaviour to precise stimulus timing and not just duration.

Author response: With reference to previous studies, we used the stimulation paradigm as per our requirements for an effective neuronal activation. Included the references. Please see the references in line 122.

As the reviewer mentioned, implanting a sniff cannula or using an implanted telemetric sensor is out of the scope of this manuscript, but we will certainly include this suggestion in our future experiments.

14) **Reviewer1:** Line 134: Please rephrase to “olfactory stimuli” or add references for other modalities.

Author response: Rephrased and added reference. Please see line 155.

15) **Reviewer1:** Line 136: Please elaborate on the direct connection between duration and olfactory identity.

Author response: Elaborated the direct connection between duration and the odor identity. Please see line 157-160.

16) **Reviewer1:** Line 138: Please rephrase to e.g. “…in response to a pattern of 10 light stimuli with a duration of 10 or 25 ms with an inter-stimulus interval (ISI) of 150 ms” to point out that it is not a just single stimulation.

Author response: Rephrased to “in response to a pattern of 10 light stimuli with a duration of 10 or 25 ms with an inter-stimulus interval (ISI) of 150 ms.”. please see line 160-163.

17) **Reviewer1:** Line 144: Point out that stimulation happens unilaterally.

Author response: Changed to Unilaterally. Line 169.

18) **Reviewer1:** Line 174: Please include references of mammalian studies to provide context for the challenge of olfactory figure-ground separation.

Author response: Included more references. Please see line 200.

19) **Reviewer1:** Figure 2, 3 & 4: The data provides the interesting finding that avoidance behaviour can consistently be evoked by the 25 ms but not the 10 ms light pulse protocol. However, there is no control or any explanation whether the 10 ms light pulse at the specific laser power used here is sufficient to activate glomeruli. The fact that the animals avoid both the safe and the light zone upon 25 ms light stimulation is striking and should be discussed in greater detail. Crucially, the authors need to heavily tone down their conclusion that “stimulus duration changes odour identity” since there is no evidence that any glomeruli are activated with the 10 ms light stimulus protocol and, thus no conclusions about odour identity can be drawn.

Author response: However we didn’t include the data in the initial manuscript, we now included additional data and figures for 10 ms positive control (Line 265-273, Fig 5 G,H,I). Our additional data shows that mice can detect 10 ms light stimulation and avoiding the light zone when trained with foot shock and 10 ms light stimulation (P = 0.0005, n=4).

20) **Reviewer1:** Line 281-283: Please rephrase, it is well-established that the number of activated glomeruli increases alongside the odour concentration.

Author response: Sentence rephrased to “It is well-established that the number of activated glomeruli increases alongside the odor concentration, making the odor generalization possible[50]” and added reference. Line 318.

21) **Reviewer1:** Line 284-285: The authors refer to the influence of flow rate on odor identification. This requires some elaboration on the mechanosensitivity of sensory neurons and projection neurons.

Author response: We added an additional paragraph explaining the influence of flow rate in odor identification. Please see line 319-325.

22) **Reviewer1:**

FIGURES
The bar graphs would greatly benefit from a clear label for baseline and light stimulation phases, e.g. by colour code or a legend

Author Response: Baseline and Light stimulation phases are mentioned in the figure legend.

23) **Reviewer1:** The dot colours representing individual animals are distracting

Author Response: We believe that it is ideal to show each animal’s responses during baseline and light stimulation. This will help the readers to understand each animal’s response pattern during the baseline and light stimulation phase.

24) **Reviewer1:** Figure 2: Point out clearly that stimulation is unilateral

Author Response: Unilateral stimulation mentioned in Figure 2 Legend.

25) **Reviewer1:** The schematic depicting the stimulation pattern is helpful but it needs clearer labelling, i.e. right and left OB.

Author Response: The schematic representation of stimulation pattern shown in Figure 2 is unilateral OB stimulation. It cannot be labelled as left or right OB, because animals in the group are either right OB stimulated, or left OB stimulated. We have mentioned it in the method section. Please see line 116-117.

**Response to Reviewer 2**

1) **Reviewer2:** My most prominent concern is whether mice are able to perceptually detect 10 ms light pulses. Two recent studies support the feasibility of the approach: Smear et al., 2011 and Li et al., 2014. While the Smear study used nearly twice as much output power, the work of Li and colleagues very nearly matches the methods used here. My concern is that the light-activated cation channel variant used in the present study (ChIEF) is less light sensitive and has a slower opening rate than ChR2 used in other studies. While I do think this point is important for the interpretation of the results in the present study, it can be addressed with added discussion and possible control data. I am curious if there is any data potentially embedded in already collected behavior videos that could help. For example, in the experiments in Figure 2, where mice show no spatial preference to the 10 ms pulses, perhaps the mice freeze, or initiate sniffing behavior when the 10 ms pulse is being delivered. This would lend confidence that OSNs are indeed being activated by the light pulses. It would also need to be shown that mice do not perform a similar behavior in the green light control experiment in Figure 6.

Author response: However we didn’t include the data in the initial manuscript, we now included additional data and figures for 10 ms positive control (Line 265-273, Fig 5 G,H,I). Our additional data shows that mice can detect 10 ms light stimulation and avoiding the light zone when trained with foot shock and 10 ms light stimulation (P = 0.0005, n=4).

We have also included a paragraph discussing the importance of ChIEF variant of ChR2 in the discussion. Please see line 334-343.

2) **Reviewer2**: Figure 3 & 4: The outcome that bilateral stimulation changes odor identity is reasonable given that different subsets of OSNs will be activated in each hemisphere. However, again, the results here do not lend confidence that mice are able to perceive the 10 ms light pulses. In Figure 3, if mice are trained on unilateral 50 ms light pulses, the test 10/50 ms pulse combination does not change the perception of the ‘odor’. At the same time, the 25/50 ms pulse combination clearly represents a different ‘odor identity’ from unilateral stimulation. A possible explanation is that the 10 ms pulses are not sufficient to activate OSNs in the contralateral bulb, while 25 ms pulses are. The same problem holds true in Figure 4 when mice are trained on bilateral pulses. In this case, if the 10 ms pulses are insufficient to activate the contralateral OSNs, it is not surprising that perception will be different - in effect, only a single hemisphere is contributing to odor identity. Again, this concern can be addressed with some behavioral readout, as mentioned in the point above. Data in Figure 5 with bilateral 10 ms pulses could be helpful.

Author response: Our experiment and previous studies suggested by Reviewer 2 (Smear et al., 2011 and Li et al., 2014) indicate that 10 ms stimulus duration is enough for activating glomeruli. Please see figure 5 G,H,I.

3) **Reviewer2:** A point that requires some clarification is whether mice are able to explore the third arm of the Y-maize, or whether the spatial location of the animal was a ‘binary’ choice between the light zone and the safe zone. Since the spatial density plots do not show the third arm, I assume this area was unavailable to the animal during testing. The supplemental videos also give this impression. With this in mind, I have concerns about the statistical comparison used. If the mouse is spending less time in the light zone, by design, it must be spending more time in the safe zone. A more appropriate comparison might be between the amount of time spent in the light zone and that same area during the baseline period.

Author response: We performed a baseline trial for each animal prior to the light activation trial. The main idea of baseline trial is to check if there is any arm preference for the animal. If the animal shows a preference to a particular arm during the baseline trial (left arm for example), we keep that arm as light zone and deliver the light when the animal entered into that arm, and the opposite arm will serve as safe zone, where there is no light stimulation. In this way we are avoiding the preferred arm as safe zone always, which eliminate spending more time in their preferred arm.

4) **Reviewer2:** In the methods please clarify whether, during the testing phase, mice were stimulated only once with light pulses for each block, or whether they were continuously stimulated each time they enter the light zone.

Author response: We have included a sentence in the manuscript “The light stimulation was delivered in the Light zone each time when the mice completely entered that zone” (line 117-118).

5) **Reviewer2:** Some discussion is missing regarding how stimulus duration might be influencing perceptual characterization of an odor. While I understand the mechanistic basis of such a phenomenon is beyond the scope of the present study, it might be worthwhile to discuss in the context of psychophysical observations. For example, it is known that long-term odor exposure decreases the perceived intensity of an odor, while the odor quality is not changed. Could it be that your mice are reporting something similar, changes in perceived intensity rather than identity?

Author response: We agree with the reviewer’s observation that long term exposure of an odor can decrease the perceived odor intensity while keeping the identity same. Our study did not include any long-term stimulation like an odor exposure, so it hardly changes the perception over the time. In this regard,

We believe that discussing the perceived odor intensity is beyond the scope of this study

Minor:

6) **Reviewer2**: For all figures with heat maps, please use the entire color scale provided. For example, in Figure 2 it appears that none of the spatial density extends into the red portion of the scale. The colors in the plots should be rescaled to utilize the entire color range. Alternatively, a new scale can be provided that more accurately reflects the range of plotted data.

Author response: Heatmaps are custom generated by the ANY-maze video tracking software for each trial which displays the amount of time mice spent in different parts of the arena. A range of colors indicates the total time spent in the area with blue indicating the shortest and the red as the longest time spend in that area. In Figure 2, mouse did not spend more time in any part of the arena. In Figure 5 B, we can see the red spot in the heat map, indicating that the mouse spend more time in that area, sitting idle. Rescaling the color plot might change the actual observation.

7) **Reviewer2:** Related to the above point, it is not clear whether the heat maps are averages of all 6 mice used, or if the data is from a single animal. If the data is from a single animal, is the same animal used in all four heat maps?

Author response: Heatmap represents single animal. We used the same animal for generating heatmaps for baseline and light activation trials.

8) **Reviewer2:** Figure 2: The amplitude of the square pluses depicted in parts C and F is different. This makes it appear that less power was used for 10 ms stimulations.

Author response: Amplitude of the 10 ms square pulse have corrected.

9) **Reviewer2:** Very minor suggestion: The y-axes of the bar plots throughout might be more meaningful if plotted as percent time rather than seconds.

Author response: We think plotting percent in the y-axis might confuse the readers about the total time of the trial.

10) **Reviewer2:** Line 143 (and throughout): Please give N values where statistics are present.

Author response: N values are added.

11) **Reviewer2:** Line 347: Li et al. is a duplicate citation.

Author response: Duplicate citation has removed.
