# Supplementary material for: The influence of stimulus duration on olfactory perception
Source: PLoS One. 2021 Jun 10;16(6):e0252931. doi: 10.1371/journal.pone.0252931 (PMC8191971; doi:10.1371/journal.pone.0252931)
Supplement: Attachment — Submitted filename: 2nd Response to reviewers.docx [file pone.0252931.s009.docx]

**PLOS ONE Response to reviewers**

**Response to the editor**

1) **Editor:** Decide if you are using “olfactory sensory neurons” or “olfactory receptor neurons” and stick with it.

Author response: Olfactory receptor neurons (ORNs) changed to Olfactory sensory neurons (OSNs)

2) **Editor:** “mice line” -> mouse line

Author response: Changed “mice line” -> mouse line

3) **Editor:** In Fig. 3C and F, do the light exposure monitors need to be exchanged?

Author response: No, light exposure arm was decided based on the baseline behavior prior to the light exposure trial. In both figures 3C and F, baseline behavior was measured separately.

**Reviewer1:** Fig. 5I: The schematic for bilateral stimulation is the same as for 5F (although squeezed horizontally) and thus of limited helpfulness without digging into the figure legend. The authors should adapt to assist the reader through the figure.

Author response: Added “Foot shock” in the Figure 5F.

**Reviewer2:** I agree with the editor's comments that calling this task a Y-maize is inappropriate given that that the task used is more analogous to a two-chamber forced choice. It is mentioned in the response to the reviewers that readers might find it confusing to change the task description; however, this reader was indeed confused by the current description as a Y-maize. I strongly encourage the authors to modify this in the text. Perhaps, in the methods, the authors could describe that a Y-maize was used to make a two-chamber task, and use “two-chamber” throughout the text.

Author response: As the reviewer 2 and the Editor suggested, we changed “Y- maze” to “two-arms maze”. We also mentioned that the “two-arms maze” was modified from Y-maze.

**Reviewer2:** I am unconvinced by the response regarding figures. It seems to be little trouble to, as Reviewer 1 suggested, add “a clear label for baseline and light stimulation phases.”

Author response: In agreement with both reviewers, we have added Baseline and Light activation in Figure 2C, which will help the reader to understand the difference between baseline and light activation trials. We believe it is not necessary to label all the figures with baseline and light activation, because the color code in all the figures are same.

**Reviewer2:** Similarly, I disagree that the full range of the color scale is being used in all figures. It appears that <25% of the range is being covered for most data. I understand that these may be software-generated figures, but the data should be optimally displayed. My suggestion is to either change the scale bar or resale the data to match the axis. The point is made in the response to the reviewers – which points out that Figure 5B is properly scaled. Why not the rest of the data?

Author response: As the Reviewer mentioned, heatmaps were generated by the software. Color scales represent the lowest amount of time (blue) and the highest amount of time (red) spend in each point. Red dot in the Figure 5 B shows that the animal spent more time in that area than the other area (like freezing). In other figures, the animal did not spend more time in one particular area but moving around. That’s the reason we see the difference between figure 5B and other figures. We also mentioned the range of color scale in the method section.

**Reviewer2:** It might be helpful to move the new control data (Figure 5G-I) to earlier in the manuscript. The question of whether the mice can detect 10 ms light pulses arises following the very first data (Figure 2) and goes unmentioned following Figures 3-4, were similar questions come up. I understand including the 10 ms data with a similar experimental approach, but this unanswered question distracts from the rest of the discussion.

Author response: We believe moving the new control data (Figure 5G-I) to earlier in the manuscript (adding it to Figure 2) will confuse the readers, because the results in the first section and figure 2 represent the single bulb activation, and the Figure 5G-I represent dual bulb activation. To avoid confusion, we think it will be ideal to add in the second part of the result section.

**Reviewer2:** Lastly a nomenclature issue. ChIEF is not synonymous with ChR2. Throughout the text ChR2 should be replaced when what is really meant is ChIEF.

Author response: ChR2 is replaced with ChIEF in appropriate places.
